# Social support reduces stress hormone levels in wild chimpanzees across stressful events and everyday affiliations

**DOI:** 10.1038/ncomms13361

**Published:** 2016-11-01

**Authors:** Roman M. Wittig, Catherine Crockford, Anja Weltring, Kevin E. Langergraber, Tobias Deschner, Klaus Zuberbühler

**Affiliations:** 1 Max Planck Institute for Evolutionary Anthropology, Department of Primatology, 04103 Leipzig, Germany; 2 University of St Andrews, School of Psychology & Neuroscience, St Andrews, KY16 9JP, UK; 3 Budongo Conservation Field Station, PO Box 362, Masindi, Uganda; 4 Arizona State University, School of Human Evolution & Social Change and Institute of Human Origins, Tempe, Arizona 85281, USA; 5 University of Neuchatel, Department of Comparative Cognition, 2000 Neuchatel, Switzerland

## Abstract

Stress is a major cause of poor health and mortality in humans and other social mammals. Close social bonds buffer stress, however much of the underlying physiological mechanism remains unknown. Here, we test two key hypotheses: bond partner effects occur only during stress (social buffering) or generally throughout daily life (main effects). We assess urinary glucocorticoids (uGC) in wild chimpanzees, with or without their bond partners, after a natural stressor, resting or everyday affiliation. Chimpanzees in the presence of, or interacting with, bond partners rather than others have lowered uGC levels across all three contexts. These results support the main effects hypothesis and indicate that hypothalamic-pituitary-adrenocortical (HPA) axis regulation is mediated by daily engagement with bond partners both within and out of stressful contexts. Regular social support with bond partners could lead to better health through daily ‘micro-management' of the HPA axis, a finding with potential medical implications for humans.

## Abstract (web summary)

The stress-reducing effects of social bonds have been hypothesized to accrue either during stressful events or across daily affiliations. Here, Wittig *et al*. show that the presence of social partners reduces levels of stress hormones in wild chimpanzees beyond stressful contexts, supporting the latter hypothesis.

For animals, including humans, there is much evidence that individuals who maintain stable, close social bonds have greater reproductive success, increased longevity and better health compared with those who do not^1^,^2^,^3^,^4^,^5^. A key mechanism through which these benefits could operate is through social buffering, in which social support provided by bond partners cushions the aversive effects of stressful events through mediation of the hypothalamic-pituitary-adrenocortical (HPA) axis^6^,^7^,^8^,^9^. In particular, the presence of bond partners during a stressful event can result in lower glucocorticoid (GC) levels^7^,^8^,^9^,^10^ or help to reduce them sooner after the event, compared with when bond partners are not available^11^,^12^,^13^.

Alternatively, bond partners may have a more generalized positive effect, insofar as subjects can rely on general social support beyond periods of acute stress. General social support may create more predictability within social interactions and more stability in the social environment^6^,^14^. This main effects hypothesis^6^ is less examined in non-human animals, despite its potential implications for promoting health and longevity^6^,^14^,^15^. Although there is some empirical support for this hypothesis in humans^15^,^16^,^17^, the underlying mechanism that enables such a main effect is unknown. Some studies suggest main effects may also be mediated by the HPA axis^6^, or by other mechanisms^14^,^17^.

Physically and psychologically-induced stress can cause dysregulation of the HPA axis, provided the stress is repeatedly acute^18^ or chronic^19^. This dysregulation can lead to severe health problems^8^,^20^, suggesting that any mechanisms that moderate such aversive effects will be under positive selection. Social buffering during stress exposure shows regulatory, stabilizing effects on HPA activity^7^,^8^, but it is equally possible that the same effect is also caused by everyday affiliations between individuals that predictably offer social support to one another.

While the main effects hypothesis predicts HPA regulatory effects of social partners in both stressful and non-stressful situations, the social buffering hypothesis excludes HPA axis regulation outside of stressful events. To test between these two hypotheses and to investigate their predicted impact on HPA axis activity, we monitored subjects' urinary glucocorticoid (uGC) levels in reaction to acute stressors, everyday affiliations and resting. Since general social support is usually measured as social integration in the community^6^,^14^ or as the presence of reliable supporters^7^,^11^,^12^,^13^, we contrasted subjects' uGC levels in the three contexts depending on the participation or presence of a bond partner, who by definition provides predictable support.

The impact of social support on stress and health is often posited as a causative relationship; however, whether social relationships promote lower stress and better health or rather whether healthier and less stressed individuals are better able to maintain relationships is not well established, given that most studies are correlational^21^. To address this issue, we used a within-subjects design, so that the same individuals were tested in each condition and we compared changes in uGC levels due to events.

Another unresolved issue is whether only active support (instrumental help)^6^,^17^ mediates HPA axis activity, or whether the mere presence of a bond partner (social companionship)^6^,^17^ is enough to do so. Also, it remains unclear whether perceived or actual support is more beneficial^14^,^17^,^22^, or whether predictability of support is a key component for stress regulation^15^,^23^.

Chimpanzees are a good model species for exploring these issues. They have highly differentiated relationships with group members, with individuals maintaining close social bonds with one or several other group members^24^. Close social bonds are defined as high rates of consistent mutual cooperative and affiliative behaviours (grooming, coalitionary support and food sharing) between two individuals, which are maintained over several months or years. Bond partners, thus by definition provide predictable social support^24^,^25^. Here, we compared the effects of partners with predictable support patterns (bond partners) with those of unpredictable support patterns (non-bond partners) on uGC levels.

In contrast to previous correlational studies, where GC measures were typically averaged over extended time periods, which included a multitude of stressful events^9^,^10^,^11^,^23^, we used an event sampling approach measuring uGC levels that corresponded to a single event^26^. Applying a fixed latency for clearance of GCs into urine^26^,^27^, we created relative uGC levels by dividing uGC levels relating to the event by uGC levels relating to before the event. Events were stressors (intergroup encounters), everyday affiliations (grooming) or control periods (resting without social interaction). Intergroup encounters are highly stressful events, associated with high GC levels^28^, in which chimpanzees face potentially lethal aggression when they encounter rival groups^29^,^30^,^31^, but they can also involve social support. A key factor in winning inter-group encounters is to out-number rivals^31^,^32^ through coordinated attacks involving coalitionary support against rivals^33^,^34^. Coalitionary and social support rather than retreat (defection) from group members is crucial to rout rivals in direct confrontations^29^,^32^. Thus, we noted the occurrence of social support during intergroup encounters, only when individuals joined in vocal or physical aggression against rivals.

To test the social buffering hypothesis^6^,^7^,^8^ in wild chimpanzees using a within-subjects design, we predict the following: if social support in chimpanzees is effective during stressors, we expected the Relationship Quality of the participants to impact on urinary glucocorticoid (uGC) levels only when experiencing a stressor. Specifically, we predicted an interaction between Event (intergroup encounter, resting, grooming) and Relationship Quality (bond, non-bond) such that uGC levels would be reduced during stressors (intergroup encounters) but not during resting or everyday affiliations (grooming), when bond partners rather than other individuals participated.

To test the main effects hypothesis^6^,^14^, we predicted the following: if social support impacts on uGC levels during everyday affiliation (grooming), resting and during a stressor (intergroup encounters), we expected the Relationship Quality of participants to impact on uGC levels irrespective of context, such that GC levels would be reduced by bond partners rather than other individuals across contexts. In addition, we predicted no interaction effects between Event and Relationship Quality.

We tested the social buffering and main effects hypotheses by comparing uGC levels in wild chimpanzees associated with three events: potentially life-threatening stressors (intergroup encounters^29^,^31^), everyday affiliations (social grooming^11^,^23^) and control periods (resting). We show that chimpanzees have reduced uGC levels when they are together with bond partners rather than other chimpanzees, irrespective of context. Further analyses revealed that the ameliorative effects of bond partners were most pronounced during contexts requiring active support rather than the passive presence of bond partners, with the largest effects during stressors then grooming contexts, and least so during resting contexts. Our results indicate that the predictable social support of bond partners may help to micro-manage HPA axis activity, during both stressful and everyday events, supporting the main effects hypothesis.

## Results

### Stressful events and everyday affiliation predict uGC levels

**Figure 1:**
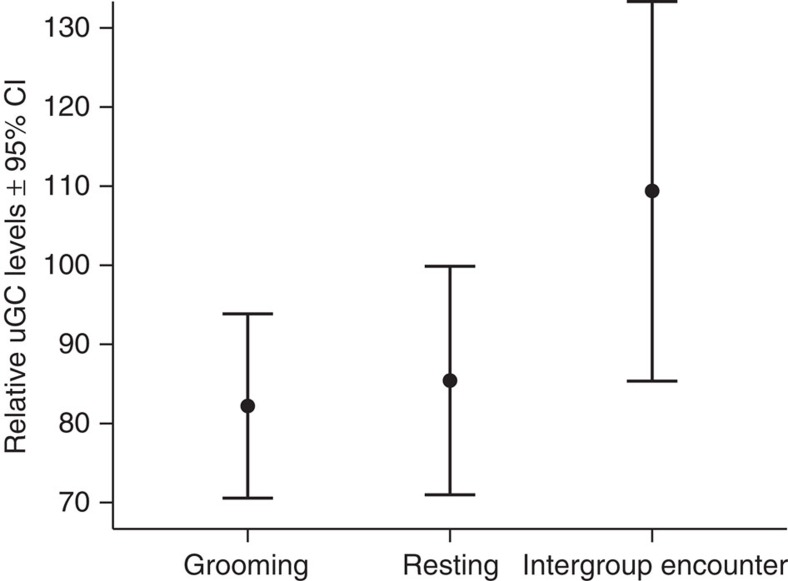
Individual relative urinary glucocorticoid (uGC) levels change with Event. Mean relative uGC levels (%)±95% confidence interval depend on the event sampled (grooming *N*=31, resting *N*=18, intergroup encounter *N*=21), such that Event predicted the relative uGC level of Sonso chimpanzees (Wald test: *df*=2, *χ2*=7.98, *P*=0.018).

We first tested if relative uGC levels were influenced by Event (intergroup encounters, resting, grooming). To control for physical exertion, security in numbers and sex effects, we added Event Duration, Number of Chimpanzees Present and Subject's Sex as control predictors. Given that subjects were sampled more than once and sometimes from the same Event, we added Subject ID and Event ID as random effects (Table 1: General Model). Both Event and Event Duration significantly affected uGC levels (likelihood ratio test: *df=2, χ2=*7.98 and *P*=0.019), but the interaction of Event and Event Duration was not significant, and therefore removed (Wald test: *df=2, χ2=*5.21, *P*=0.074, Supplementary Table 1). Intergroup encounters resulted in higher relative uGC levels than grooming or resting events (Fig. 1; Table 1: General Model) and longer events resulted in higher relative uGC levels (Supplementary Fig. 1), while Number of Chimpanzees Present and Subject's Sex did not significantly influence uGC levels. The effect size of the fixed effects in model 1 was *R*^2^=0.170.

### Social buffering and main effects hypotheses

**Figure 2:**
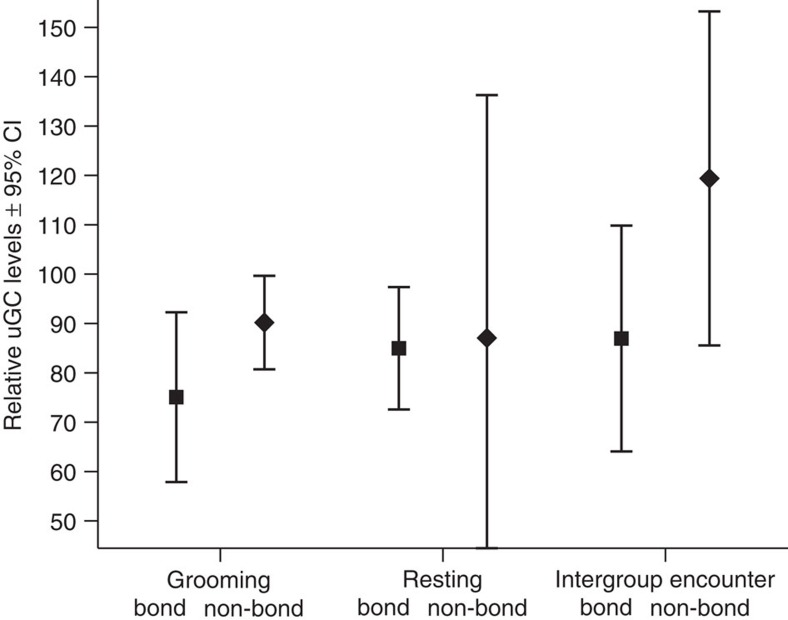
Event and Relationship Quality change individual relative urinary glucocorticoid (uGC) levels. Mean relative uGC levels (%)±95% confidence interval depend on the Event sampled: occurring with either a bond or a non-bond partner (grooming with bond partner *N*=14 and non-bond partner *N*=17, resting with bond partner *N*=13 and with non-bond partner *N*=5, intergroup encounter with bond partner *N*=11 and non-bond partner *N*=10). Both Event (Wald test: *df*=2, *χ2*=6.14, *P*=0.047) and Relationship Quality (Wald test: *df*=1, *χ2*=6.77, *P*=0.009) predicted the relative uGC level of Sonso chimpanzees.

In the second model we investigated whether the social buffering or main effects hypotheses were best supported by the data. We tested if relative uGC levels were affected by the interaction of predictors Event and Relationship Quality (bond versus non-bond partners), while controlling for the Sex and Kin (close maternal kin) relationships of the two participants, and including Subject ID and Event ID as random factors. Although the full model was significantly different from the null model (likelihood ratio test: *df*=5*, χ2*=13.93*, P*=0.016), we neither found a significant interaction between Event and Relationship Quality on uGC levels, nor between Subject's Sex and Partner's Sex (Table 2a: Social Buffering Model, Fig. 2).

After removing both non-significant interactions from the model, both test predictors (Event and Relationship Quality) significantly influenced relative uGC levels (Table 2b: Main Effects Model, Fig. 2). Specifically, relative uGC levels were on average almost 22% higher after intergroup encounters than after grooming events and on average >8% higher after resting than grooming events. In addition, subjects participating in events with bond partners rather than other individuals had on average 23% lower relative uGC levels, across events (Table 2b: Main Effects Model). Neither Kin nor Sex of subject showed a significant influence on relative uGC levels, while the relative uGC levels varied depending on the Sex of Partner, with subjects of male partners having higher relative uGC levels. The effect size of the fixed effects in model 2 was *R*^2^=0.196.

### Extent of bond partner impact on uGC levels

Engaging with bond partners lowered relative uGC levels across all events, although to determine the extent of bond partner impact within contexts requires further testing. Therefore, using bootstrapping, we tested whether chimpanzees' relative uGC levels within each context were significantly higher or lower than expected levels. We calculated the expected relative uGC level by estimating the diurnal decline in uGC levels (Supplementary Note 1, Supplementary Fig. 2). The results of the bootsrap procedure showed that grooming with a bond partner decreased uGC levels (bootstrap: mean deviation±SE=−13.3±6.5, CI=(−25.9; −0.8), *N*=10, *P*<0.05) and engaging in intergroup encounters without a bond partner increased uGC levels (bootstrap: mean deviation±SE=31.9±12.9, CI=(7.9; 64.9), *N*=7, *P*<0.05). In contrast, when grooming a non-bond partner (bootstrap: mean deviation±SE=2.7±4.1, CI=(−6.4, 9.5), *N*=11) and engaging in intergroup encounters with a bond partner (bootstrap: mean deviation±SE=−0.5±9.0, CI=(−17.8; 16.5), *N*=8), confidence intervals included 0 and therefore were not different from expected relative uGC levels. Finally, relative uGC levels after resting with a bond partner did not differ from expected values (bootstrap: mean deviation±SE=−2.5±5.5, CI=(−13.3; 7.0), *N*=13), but resting without bond partners had an insufficient sample size to be tested (*N*=5).

## Discussion

Within each event, subjects' relative urinary glucocorticoid levels were lower when engaging with bond partners rather than other individuals, whether during a stressor (intergroup encounters), everyday affiliation (grooming) or resting. Bond partner effects, however, were strongest during intergroup encounters, then during grooming and least during resting. The social buffering hypothesis predicts bond partner effects during stressors only, whilst the main effects hypothesis expects bond partner effects during both stressors and everyday events. Thus, our results show support for the main effects rather than the social buffering hypothesis (see Fig. 2 in ref. 6).

Chimpanzees showed significantly higher relative uGC levels after stressful events (intergroup encounters) compared with after resting. However, uGC elevation was evident only when bond partners did not participate in the encounter. The same chimpanzees facing potentially life-threatening intergroup encounters together with a bond partner did not have elevated uGC levels. This result demonstrates that the participation of a bond partner, who by definition provides predictable social support, regulates HPA activity and buffers the stress reaction even in potentially lethal situations. Less predictable supporters were less effective in buffering uGC levels. Our results showing buffering effects of bond partners on HPA axis activity concur with experimental studies in humans^12^,^13^, where bond partners buffer psychologically-induced stress, and studies examining naturally occurring social stressors in non-human primates^9^,^10^,^11^. In the non-human primate studies, correlations show that bond partners seem to buffer baseline GC levels following severe social stressors, such as, the threat of infanticide^11^, sudden social isolation^10^ and during milder social stressors, such as conspecific aggression^9^.

Chimpanzees showed lower relative uGC levels during everyday affiliative interactions (grooming events) compared with resting events. However, uGC levels decreased only when chimpanzees groomed with a bond partner. In contrast, subjects grooming with a non-bond partner showed uGC levels similar to resting control samples. This shows that bond partners have moderating effects on HPA activity during everyday grooming events, outside of stressor contexts. A correlational study on wild baboons is also suggestive of this pattern, where female baboons during periods of social stability had lower baseline GC levels when they were able to focus their grooming on a few preferred individuals rather than showing more distributed grooming patterns^23^. In both stressful and grooming contexts, our results indicate a positive relationship between predictable social support objectively measured from social interaction histories and the stress buffering potential of supporters.

Model 2 suggests that bond partner effects on uGC levels were also evident during resting events. This result, however, has to be treated with caution, since sample size for resting without bond-partners is low which leads to instability problems. Nevertheless, the result is interesting since experimental studies with humans usually do not distinguish between social support due to the mere presence of bond partners^12^, and active support offered by bond partners^13^. In our study, although the effect is the same, the estimates due to bond partners are different (Fig. 2), increasing from resting to grooming to intergroup contexts. This finding indicates that active support from bond partners is more effective in lowering uGC levels than is their mere presence.

Everyday affiliative interactions with bond partners have been predicted to improve physical and mental health, although the mechanism through which the benefits might be accrued has remained evasive^14^,^16^. Cohen and Wills^6^ suggested that benefits might be mediated through similar mechanisms as social buffering effects, specifically involving the HPA axis. Our results suggest that this is indeed the case: engaging in three types of activity with predictable bond partners lowered subjects' uGC levels, whether during highly stressful interactions, resting or everyday affiliative interactions.

Given that both male and female subjects participate in grooming and intergroup encounters^34^ (and this study), we contrasted the relative uGC levels of both female and male subjects, as well as the sex combination of the dyad. Neither subjects' sex nor the sex combination of the dyad influenced relative uGC levels in any model. Thus, for both male and female chimpanzees, the impact of participating in stressors or everyday affiliations with a predictable partner of either sex, compared with another individual, similarly regulates the HPA axis. This result is not unexpected, even in intergroup encounters, where both males and females participate. Females engage often in vocal aggression^34^ (Supplementary Movie 1), which is highly effective in deterring within-group^24^, as well as out-group members^34^,^35^. Both sexes can incur high costs from attacking or being attacked (injury or death)^31^,^36^,^37^. However, it is likely that males do not gain comparable vocal or physical support from females as they do from males, and we cannot rule out that a larger sample may show some sex effects. It should be noted that in this data set, all mixed-sex bond partner dyads consisted of mothers and their sons.

For grooming events, the uGC patterns mirror urinary oxytocin levels found after grooming in the same chimpanzee population^38^. In this previous study, grooming with a bond partner was associated with higher urinary oxytocin levels compared with grooming with a non-bond partner or periods of resting, independent of whether subjects were giving or receiving grooming. These contrasting GC and oxytocin results are similar to those of two social buffering experiments exposing humans to a psychological stressor. In trials in which social support was offered from a bond partner, subjects had earlier decreasing GC levels. One study showed lower GC levels and returned faster to baseline in conditions in which urinary oxytocin levels increased^13^. The other study showed greatest GC decreases in trials with both social support by bond partners and intranasal application of oxytocin before the stressor^12^. Thus our observations are in line with the idea that oxytocin has a dampening role in cortisol secretion^7^,^8^,^9^. In chimpanzees, active social support from bond partners seems to have more pronounced effects on urinary oxytocin levels^38^, as well as on uGC levels, than the mere presence of bond partners^38^. Likewise, it is possible that participating in activities with predictable bond partners in humans may be more effective in HPA axis management than either merely associating with bond partners without active participation, or participation with less predictable supporters. To date, these conditions have rarely been contrasted in the human literature^12^,^13^. Given that we examined the same chimpanzees in the same contexts with and without bond partner participation, and that we measured relative change in uGC levels after, compared with before, the events, supports a causative effect between bond partner support and lower HPA axis activity, in everyday affiliations as well as during stressors. The neuropeptide oxytocin may be key to the underlying mechanism precipitating the social regulation of the HPA axis^7^,^8^,^9^.

Crucially, engaging an oxytocin—HPA axis interaction by participating with predictable supporters during ordinary everyday affiliative contexts may help maintain a healthy homoeostasis. We propose that a possible underlying process could be that daily affiliative social interactions help regulate—or micromanage—the HPA axis. This in turn may help to maintain stable immune-function, cardio-function, fertility, cognition and mood, key aspects known to be affected by a dysregulated HPA axis^8^,^14^,^15^,^39^. The close phylogenetic relationship between humans and chimpanzees suggests that HPA axis micro-management through daily affiliative social interactions could also occur in humans.

Bond partner effects were strong such that even during a potentially life-threatening inter-group encounter, bond partners buffered the stress response. It could be argued that since the triggering of the stress response is vital for maximizing the physical response in a fight situation^40^, it is not necessarily adaptive for bond partners to buffer the stress response in fight contexts. However, bond partners by definition in this study have a predictable history of providing social and agonistic support to one another^24^. Thus, it may be that subjects are indeed safer when engaging in inter-group encounters when bond partners are also participating and subjects thus ‘require' less or shorter HPA axis activity to ensure survival. This interpretation might offer one explanation as to why chimpanzees and humans seek bond partners when initiating conflict with rival groups^30^,^33^,^36^. Another possible explanation for these results is that our method can only measure strong changes in relative uGC levels and do not detect short or minor increases in stress response activity.

In this study, chimpanzees supported by bond partners showed no significant up-regulation of their HPA axis when participating in an intense stressor (inter-group encounters), and showed significant down-regulation of their HPA axis in everyday affiliations (grooming) compared with support from other individuals. These results suggest that bond partners can influence HPA axis activity both during highly stressful situations, as well as during everyday activities, such as grooming. The HPA axis is vulnerable to dysregulation, particularly when exposed to intense or repeated stressors^8^,^18^,^19^,^20^. Such HPA axis dysregulation can have negative and long-term effects on physical and mental health^8^,^18^,^19^,^20^,^39^,^41^. Social buffering may have an immediate effect during a stressor of reducing HPA axis activity^6^,^7^,^8^,^12^,^13^. However, daily management of the HPA axis through repeated affiliations from predictable social supporters may protect against dysregulation of the HPA axis over the longer term^6^. The mechanism that might account for reported effects of better health when engaging in everyday affiliations with bond partners, has remained elusive^6^,^14^,^16^,^17^,^21^. Here, we demonstrate that, as for stressors, bond partners also downregulate HPA axis activity even during an everyday affiliation, grooming and with a weaker effect, by their mere presence when resting, as proposed by Cohen and Wills' main effects hypothesis^6^. From these results, we hypothesize for future testing that regular and repeated, everyday affiliations have the potential to regularly and repeatedly re-align the HPA axis throughout the day. This could amount to ‘micro-managing' effects on the regulation of the HPA axis, resulting in stable HPA activity over time, and providing long-term benefits to physical and mental health.

## Methods

### Subjects

We observed chimpanzees (*Pan troglodytes schweinfurthii*, Supplementary Data 1) of the Sonso community, Budongo Forest (1°35′—1°55′ N, 31°08′—31°42′ E), Uganda, from February 2008 to July 2010. The Sonso community has been continuously observed since 1990 (ref. 42), and was comprised of 15 males (adults: ≥15 years: 10; subadult: 10–14 years: 5), 35 females (adult: ≥14 years: 27; subadult: 10–13 years: 8) and 28 juvenile and infants during the study period. We sampled urine from nine adult male and eight adult female chimpanzees—those that were regularly observed to improve the likelihood of within-subject sampling across behavioural conditions. Permissions to conduct this research was granted by Uganda Wildlife Authority (TDO/33/02) and Uganda National Council for Science and Technology (NS 181) and overseen by Budongo Conservation Field Station and the University Teaching and Research Ethics Committee of St Andrews University.

### Observation methods

A team of up to six observers followed up to three parties of chimpanzees (independently moving subgroups of flexible composition) from ∼7 a.m. to 5 p.m. through the forest, recording behaviours using ‘all occurrence' sampling^43^ for aggressive (agonistic support, contact and non-contact aggression) and affiliative social interactions (grooming, food sharing, sitting in close proximity (within 1 m)), and 15 min scan sampling for party composition, where ‘party' is a subgroup of individuals in view. In addition, we recorded intergroup encounters whenever they were observed. We focused on three event types: (1) intergroup encounters: subjects had auditory or visual contact with a rival community. Subjects were sampled if classified as engaging in social support of group members, which required approaching in the direction of the rivals and responding with chorused (coalitionary) vocal aggressive behaviour (barks, pant roars, buttress drumming and pant hoots) and/or coalitionary physically aggressive behaviour (charging, chasing, hitting and biting) towards out-group members. Support was coded as ‘mutual' in all cases, given that all receive a benefit from another's participation, whether it occurs before or after their own. These intergroup events were also not characterized by grooming bouts either before, during or after the intergroup encounters. Since the natural frequency of encounters with the rival communities was low (nine encounters were sampled during the observation time), we also conducted four drumming experiments to simulate the presence of a rival community (see experimental procedure); (2) resting events, in which one individual had no social interaction for a minimum of one hour and was sitting or lying for at least the first 30 min; and (3) grooming events, in which two individuals groomed each other for at least 20 min without a break and had no additional social interaction for at least 1 h after the beginning of the grooming event. Grooming duration as well as minutes spent giving, receiving or engaging in both simultaneously (mutual) were recorded.

### Experimental procedure

We waited for a subgroup—or party—of chimpanzees to rest in the periphery of their territory in an area where we had observed intergroup encounters during the last year. After resting without vocalising for at least 10 min, an experienced field assistant mimicked a typical mid-length chimpanzee buttress tree drum, striking 7 beats with his fists on the buttress roots of a tree about 100 m away from the resting party in the direction of the rival community's territory. Importantly, before and during the drumming simulation, we took extreme care that Sonso chimpanzees could not see or hear any person moving in the direction where the drumming experiment would or just had occurred. In all four trials, the chimpanzees showed behaviour indistinguishable from natural intergroup encounters (pilo-erection, reassurance behaviours, cautious and silent approach to the drumming tree followed by aggressive displays, aggressive vocalizations, like pant-roars and pant-barks and drumming). Eight of 21 intergroup encounter data points were experimentally simulated encounters (Supplementary Data 1). We found that experimentally simulated intergroup encounters produced no different reaction in uGC levels than natural encounters (likelihood ratio test: *χ*^2^=0.869, *df*=2, *P*=0.648; Supplementary Table 2). Therefore we merged natural observations and experimental simulations of intergroup encounters.

### Urine collection and hormone data analysis

After observing one of our target chimpanzees engaging in one of the three events (grooming, resting, intergroup encounters), we switched to ‘focal animal' sampling^43^ of that individual for the next 6 h, collecting every possible urine sample and recording each behaviour and change in activity (see ref. 26). Urine was either pipetted from plastic bags, after the bags were tied over a forked stick and held in the urine stream when subjects were sitting in a tree, or from leaf matter when urination occurred on the ground after subjects had moved away. After collection, urine samples were stored in a thermos flask containing ice and were frozen in liquid nitrogen upon arrival in camp, which was within 10 h after collection. Urine collection did not commence if subjects had engaged in aggression or grooming within the hour before the activities, and was aborted if subjects engaged in additional aggression or grooming within two hours after the activity. We collected a total of *N*=574 urine samples from adult chimpanzees where we were able to determine uGC levels.

We were able to assign *N*=394 of these urine samples (nine male and eight female chimpanzees, Supplementary Data 1) to a single event, either: (1) social grooming (social support in an everyday context), sustained for >20 min (*N*=31 data point including 172 urine samples in 28 events, *N*=14 subjects); (2) resting (no support control), sustained for >30 min (*N*=18 data points including 108 urine samples in 18 events, *N*=16 subjects); and (3) intergroup encounters (social support in a stressful context), when chimpanzees saw out-group chimpanzees or reacted vocally to calls of chimpanzees from real or simulated intergroup encounters (*N*=21 data points from natural encounters including 73 urine samples in 9 events, *N*=7 subjects; and from simulated encounters including 41 urine samples in 4 events, *N*=6 subjects). Chimpanzees show intimate reassurance behaviour after detecting rival groups, including embracing, placing their finger in others' mouth, holding others' testes or grooming (Supplementary Movie 1).

Urinary GC levels were measured at the Behavioural Endocrinology Lab at the Max Planck Institute for Evolutionary Anthropology using high-performance liquid chromatography-tandem mass spectrometry (LC-MS/MS)^44^. Samples with a recovery of the internal standard deviating by <±50% from the expected value were included in the analysis. In cases of large deviation we re-measured the samples. If a large deviation persisted, we re-extracted and re-measured the samples. We excluded samples where the large deviation still persisted. Examination of LC-MS/MS data was carried out with MassLynx (version 4.1; QuanLynx-Software). Only a fraction of plasma cortisol can be found in chimpanzee urine^27^, while metabolites of cortisol are found in higher quantities^44^. To quantify the urinary glucocorticoid excretion (uGC), we used the sum of urinary cortisol plus four of its metabolites (tetrahydrocortisol, tetrahydrocortisone, 5β-androstane and 11-oxoetiocholanolon). The sum of uGC comprised on average 9% cortisol, 37% tetrahydrocortisol, 35% tetrahydrocortison, 5% 5β-androstane and 14% 11- oxoetiocholanolon. We corrected the uGC levels with the creatinine levels of each sample to control for differences in water content of urine samples^45^. We excluded samples with a creatinine level of <0.05 mg creatinine ml^−1^ urine from the analysis.

Using an event-sampling approach^26^,^38^,^46^, we then calculated the relative uGC levels for each Event, using a method that took the clearance window of cortisol into urine into account (see Fig. 1 in ref. 26). We defined a pre-period (representing the uGC levels from before the event) and a peak-period (representing the uGC levels related to the event), taking into account the delayed GC clearance in chimpanzee urine^26^,^27^ and the diurnal decrease of uGC levels in chimpanzees^47^ (Supplementary Fig. 2, Supplementary Note 1). We calculated the mean uGC levels for the pre-period (from samples taken between the start of the event until 135 min after the start of the event) and the mean uGC levels for the peak-period (from samples taken between 135 min until 270 min after the event + event duration) for each event. Samples taken within 30 min after the pre or the peak-period were still assigned to the preceding period, if the focal individual had not urinated within the last 30 min of the preceding period^26^. Finally we calculated the relative uGC levels as a percentage of how high the uGC levels were during the peak-period compared with the pre-period:


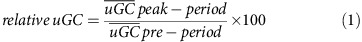


We calculated an average decrease of 5.55% of the uGC level per hour of the day. Taking an average time difference of 135 min between peak and pre-period samples, peak-period samples should be 12.49% lower than pre-period samples. A relative uGC level of about 87.5% therefore reflected a neutral effect of the event behaviour on the uGC level only including the estimated diurnal decline of uGC. Levels above the expected relative uGC level of 87.5% would represent an increase and below the 87.5% would represent a decrease in uGC levels due to the event.

### Definition of social bonds

The Relationship Quality of chimpanzees can be defined by three components^48^ such that those that share close social bonds engage in: (1) high rates of socio-positive and cooperative behaviours and low rates of socio-negative behaviours, (2) balanced or symmetrical offering of high rates of socio-positive and cooperative behaviours over time, (3) sustained patterns of (1) and (2) over at least a six month period. We described the quality of social relationships by calculating the composite relationship index^24^,^38^,^46^ (CRI) over quarter-year periods for all possible dyads. The CRI contrasts socio-positive and negative behaviours in relation to average behaviour rates per sex combination across dyads.


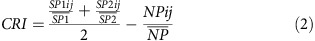


where *SP*1=rate of grooming bouts plus rate of resting in 1m proximity; *SP*2=rate of food sharing plus rate of coalitionary support; *NP*=rate of aggression and contra-intervention, *i*=individual (receiver of service) and *j*=dyad partner (offering of service). A positive CRI indicated a more socio-positive relationship and a negative CRI showed a socio-negative relationship. A good indication that grooming and food-sharing are important bonding behaviours is that both have been linked with elevated urinary oxytocin levels in chimpanzees^38^,^46^. Dyads were only defined as bond partners when socio-positive relationships were symmetrical (reflecting similar rates of offered affiliations from both dyad partners) and long lasting (≥ 6 months: at least two consecutive blocks of quarter-year periods). Balance and duration of relationships: scored through either a mutual socio-positive relationship (CRI>0) during the annual quarter of the sampled event and the preceding quarter, or a large mutual socio-positive relationship (CRI>10) during one of the quarters and a socio-neutral or positive relationship (CRI≥0) during the other quarter^24^,^38^,^46^.

Bond partners were defined as those with whom subjects engaged in high rates of affiliative and cooperative behaviours (grooming, food sharing, coalitionary support) and low rates of aggression in both directions over at least two consecutive three month time periods providing an objective measure of historical predictability of social support^24^. A histogram of mean CRI scores per dyad shows a bimodal distribution, with bonded relationships clustering in the higher scores and non-bonded relationships clustering around zero (Supplementary Fig. 3). For this reason, we use Relationship Quality as a binomial variable.

### Definition of kin relationships

We used genetic analysis to classify dydas as close maternal kin (that is, mothers with offspring and maternal siblings) or not. Previous research suggests that as in most other group-living primates^49^, chimpanzees can recognize and discriminate their mothers and maternal siblings^25^. However, the ability of chimpanzees to recognize more distant maternal kin, who anyways would be rare when all males stay in their natal group and the vast majority of female disperse, as occurs in Sonso, has not been demonstrated. Nor do chimpanzees seem to recognize and discriminate their paternal kin^25^. We collected fresh faecal samples after individually identified chimpanzees were observed defecating. Samples were either collected in plastic tubes and frozen within a 12 h period, collected in plastic tubes containing dessicating silica beads or collected in tubes filled with 95–99% ethanol and then transferred to tubes containing dessicating silica beads after 12–24 h (ref. 50). DNA was extracted from faeces using the Qiagen DNA stool kit. We genotyped 92 chimpanzees at up to 19 autosomal microsatellite loci. To guard against allelic dropout at the autosomal loci, we first estimated the amount of amplifiable DNA in each extract through quantitative PCR, and then followed previously established guidelines showing that depending on the amount of amplifiable DNA present in an extract, up to four independent PCR replications are required to be 99% confident that a putatively homozygous genotype is indeed homozygous^51^. For heterozygous genotypes, each allele was observed in at least two independent PCRs (ref. 51). To guard against misidentification of individuals during sample collection or sample mix-up, in dyads that we strongly suspected from behavioural observations to be mother-offspring pairs we checked that individuals shared at least one allele at every autosomal locus. For individuals without suspected first-order maternal relatives, we used a second, independently collected faecal sample to repeat the genotyping at six or more loci and required a perfect match with the original genotype. Using these methods, we were able to determine that <1% of samples came from an incorrectly identified individual. In these cases, we genotyped additional extracts from independently collected faecal sample until the conflict was resolved. Microsatellite genotypes were produced through a two-step PCR multiplex procedure, using unlabelled primers for all 19 primers in the first amplification, and fluorescently labelled forward primers and nested primers in the subsequent single locus PCR reaction^51^. PCR products were electrophoresed on an ABI PRISM 3100 Genetic Analyzer. Allele sizes determined using an internal size standard (ROX labelled HD400) and Genemapper software version 3.7 (ref. 51).

We used the autosomal genotypes in likelihood based maternity analyses^52^. All eight dydas suspected of being mother-offspring from behavioural observations shared an allele at each autosomal microsatellite locus and had maternity assignments at the 95% confidence level using the CERVUS likelihood based parentage program (Supplementary Data 2). There were two dyads (Harriette—Gladis and Kalema—Kutu), where both members of a pair had unassigned mothers, because the mothers' samples were unavailable for genotyping; for these two dyads the maternity assignments precluded a mother-offspring but not a maternal sibling relationship. However, for one dyad (Kalema—Kutu) we were able to exclude the possibility that they were maternal siblings because they had different 473 base pair haplotypes at the hypervariable region-1 of the maternally inherited mitochondrial DNA (ref. 53). While we could not use genetic data to exclude the possibility that the other dyad (Harriette—Gladis) were maternal siblings, we consider this unlikely given the low incidence of female philopatry at Sonso and in chimpanzees more generally (suggesting that Harriette and Gladis are immigrants rather than natal to Sonso), as well as research in another community showing that it is extremely rare for maternal sisters to transfer from their natal community to the same new community^54^. We thus classified Harriette—Gladis as unrelated in all analyses.

### Statistics

We employed linear mixed models^55^ to test the effects of the test predictors Event (Model 1) and Event together with Relationship Quality (Model 2) on relative uGC levels. We included Subject Identity and unique Event Identity as random factors and included several control predictors as additional fixed effects. To control for ‘safety in numbers' we included the Number of Chimpanzees Present during each event. To control for expected glucocorticoid release due to energetic costs during intergroup encounters^56^, we included the event duration, predicting an interaction with event and event duration such that longer intergroup encounters should result in higher energetic output and thus higher uGC levels than shorter ones. This is in contrast to grooming and resting contexts in which no increase in energetic output was expected with event duration. To control for effects related to either subject's sex, or partner's sex or the combination of both, we included an interaction of these terms. Finally, when testing the Relationship Quality we controlled for kin, since we expected close social bonds would provide uGC level effects regardless of kinship.

The models were fitted in R 3.1.3 (ref. 57) using the function ‘lmer' of the R-package lme4 (ref. 58) with Maximum Likelihood. Residuals were normally distributed and homogenous, as shown by visual inspection of qq plots and residual plots against fitted values. We checked for model stability by excluding subjects one at a time from the data and found models were stable, apart from model 2a (Social Buffering Model) where the interactions of Event and Relationship Quality, as well as the interaction of Subject's and Partner's Sex showed some instability and thus requires careful interpretation. To investigate whether or not the skewed distribution in the resting event has caused the insignificant effect for the test predictors' interaction term, we run a full versus null model comparison, testing the interaction of Event and Relationship Quality only for resting events and intergroup encounters (Supplementary Table 3). This comparison showed no significant effect of the interaction term (Wald test: *df=1, χ2*=1.01, *P*=0.315), confirming the result of model 2a. Variables did not exhibit problems of collinearity (Variance Inflation Factor <4 in all cases, derived using R-package car^59^, and were tested using a standard linear model excluding the random effect), suggesting that each predictor variable accounted for a portion of the variance. We established the significance of the full model as compared with the null model (comprised of control predictors and random factors) using a likelihood ratio test^60^. Since interactions between predictors do not allow interpretation of the effect of the respective fixed factors in the model, we removed non-significant interactions to investigate the effects of the predictors. We calculated the marginal *R*^2^ (relative variance explained by fixed factors in relation to total variance) as the effect size for the model.

Finally, we used bootstrap sampling methods^61^,^62^ with 1,000 permutations to test whether relative uGC levels were significantly smaller or larger than the expected relative value, which was calculated to include estimated diurnal decline^26^,^47^ (Supplementary Note 1). We calculated the mean for each individual for each event with or without bond partner engagement and calculated the deviation from the expected value (expected relative uGC=87.5%). Values above 0 indicated an increase of uGC levels due to the event, values below 0 indicated a decrease of uGC levels due to the event. Since we had directed predictions (increase of uGC when interacting with a non-bond partner, decrease of uGC levels when interacting with a bond partner), we calculated the 5% interval on the predicted side of deviation from the expected value.

### Data availability

The authors declare that the data supporting the findings of this study are available within the paper and its Supplementary Information files.

## Additional information

**How to cite this article:** Wittig, R. M. *et al*. Social support reduces stress hormone levels in wild chimpanzees across stressful events and everyday affiliations. *Nat. Commun.* **7,** 13361 doi: 10.1038/ncomms13361 (2016).

**Publisher's note:** Springer Nature remains neutral with regard to jurisdictional claims in published maps and institutional affiliations.

## Supplementary Material

Supplementary Information Supplementary Figures 1-3, Supplementary Tables 1-3 and Supplementary Note 1

Supplementary Data 1 Dataset used in the model fitting

Supplementary Data 2 Level of genetic relatedness between interaction partners

Supplementary Movie 1 Footage filmed during different intergroup encounters between different chimpanzee communities in the Tai National Park, Côte d'Ivoire. Copyright Tai Chimpanzee Project, filmed by Liran Samuni. 00:00 - 00:21 Recruiting individuals for a patrol, 00:21 - 00:34 Silently walking in line towards the periphery of the territory or where they have heard neighbours (males and females), 00:34 - 00:50 Detecting traces of the neighbours, sniffing the ground (males and females), 00:50 - 01:08 Close to making visual contact with the neighbours, the party stops repeatedly, 01:08 - 01:18 Contact - the chimpanzees attack the neighbours, 01:18 - 01:42 Counter attack by the neighbours - including females, 01:42 - 02:12 Auditory combat with pant-hoots, screams, barks and drums - including females.

## Figures and Tables

**Table 1:** The impact of Event on urinary glucocorticoid (uGC) levels.

Response variable:	relative uGC		*P* value	Parameter	*Estimate*	*SE*	*t*
Predictor variable	*df*	*χ2*					
*General model*							
				Intercept	65.8	11.7	
Event*	**2**	**7.98**	**0.018**	**Intergroup**	**25.55**	**9.36**	**2.73**
				**Rest**	**−4.50**	**9.35**	**−0.48**
				**Groom**	**0**		
** **Subject's Sex†	1	0.51	0.474	Male	−6.17	8.33	−0.74
** **Number of Chimpanzees Present†	1	0.05	0.828		0.15	0.67	0.23
Event Duration†	**1**	**7.60**	**0.006**	**Minutes**	**0.45**	**0.15**	**2.86**

Random factors: Identity of Subject, Event. Bold: *P*<0.05. Intergroup: Intergroup encounter, Rest: resting event, Groom: grooming event. Likelihood ratio test (full versus null model comparison): *df=2, χ2=*7.98, *P*=0.019. Effect Size of fixed effects: marginal *R*^2^=0.170. This model with the non-significant interaction of duration and event is presented in Supplementary Table 1.

^*^LMM: *test predictor,

^†^control variable.

**Table 2:** The impact of Event and Relationship Quality on urinary glucocorticoid (uGC) levels.

Response variable:	relative uGC		*P* value	Parameter	*Estimate*	*SE*	*t*
Predictor variable	*df*	*χ2*					
(a) *Social Buffering Model*							
				Intercept	68.01	16.13	
Event × Relationship Quality*	2	1.66	0.437	Intergroup × Bond	−13.71	16.64	−0.82
				Rest × Bond	13.80	19.55	0.71
				Groom × Bond	0		
Kin†	1	2.29	0.130	Kin	20.32	13.15	1.55
Subject's Sex × Partner's Sex†	1	0.16	0.686	Male × Male	−8.01	19.77	−0.41
(b)*Main Effects Model*							
				Intercept	71.43	14.78	
Event*	**2**	**6.14**	**0.047**	**Intergroup**	**21.78**	**8.60**	**2.53**
				**Rest**	**8.35**	**9.37**	**0.89**
				**Groom**	**0**		
Relationship Quality*	**1**	**6.77**	**0.009**	**Bond**	**−23.13**	**8.65**	**−2.67**
Kin†	1	2.78	0.096	Kin	20.99	12.27	1.71
Subject's Sex†	1	0.01	0.979	Male	−0.25	9.66	−0.03
Partner's Sex†	**1**	**4.46**	**0.035**	**Male**	**21.77**	**10.13**	**2.15**

Random factors: Identity of Subject, Event. Bold: *P*<0.05. Intergroup: Intergroup encounter, Rest: resting event, Groom: grooming event. (a) Social Buffering Model testing social buffering hypothesis. Likelihood ratio test (full versus null model comparison): *df=5, χ2*=13.93, *P*=0.016. (b) Main Effects Model testing the main effects hypothesis. Likelihood ratio test (full versus null model comparison): *df=3, χ2*=12.94, *P*=0.005. Effect Size of fixed effects: marginal *R*^2^=0.196.

^*^LMM: *test predictor,

^†^control variable.
