# Supplementary material for: Social support reduces stress hormone levels in wild chimpanzees across stressful events and everyday affiliations
Source: Nat Commun. 2016 Nov 1;7:13361. doi: 10.1038/ncomms13361 (PMC5097121; doi:10.1038/ncomms13361)
Supplement: Supplementary Information — Supplementary Figures 1-3, Supplementary Tables 1-3 and Supplementary Note 1 [file ncomms13361-s1.pdf]

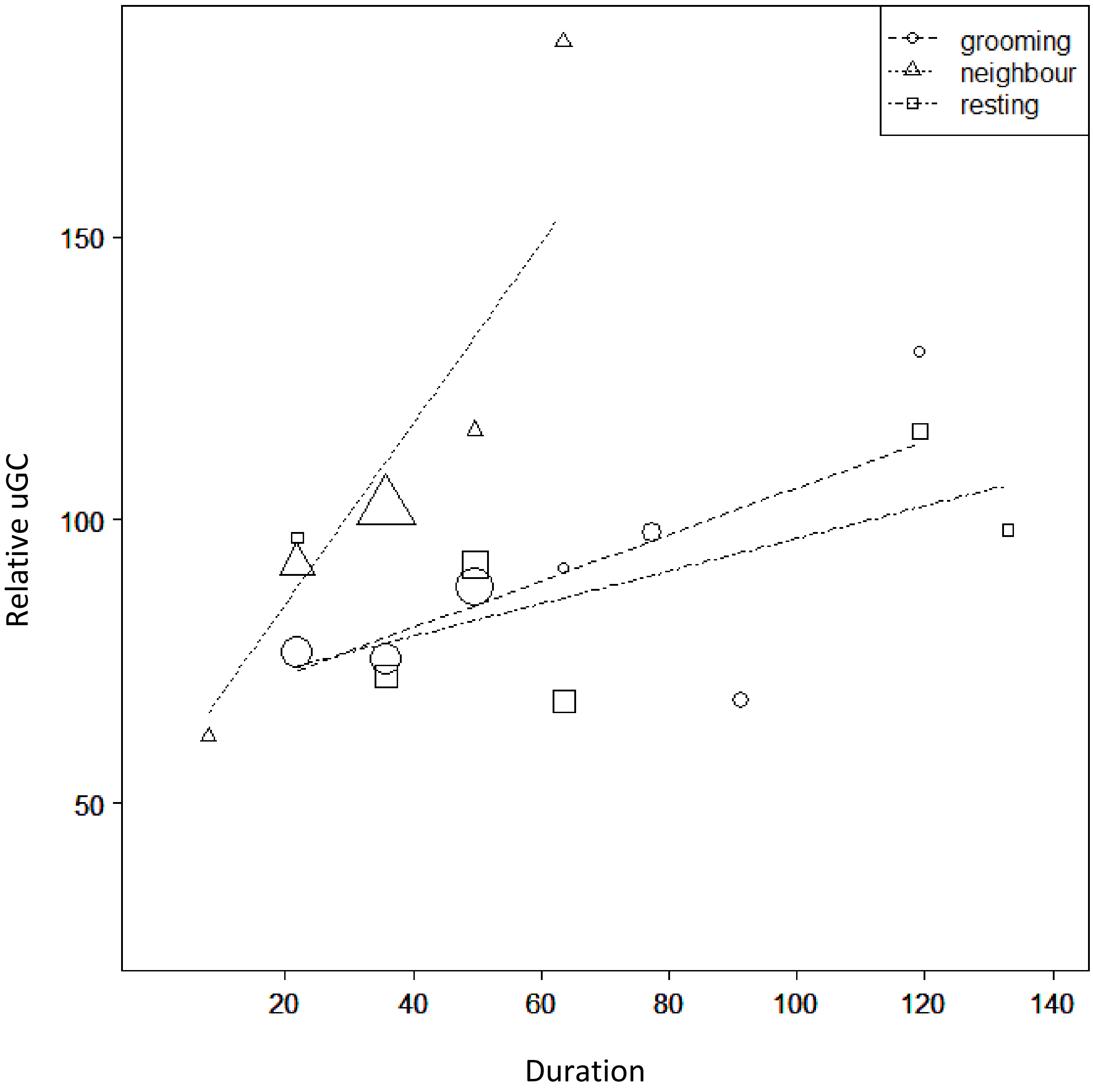

<u>Supplementary Figure 1:</u> Relative urinary glucocorticoid levels and the interaction of Event and Duration of Event. Relative uGC levels [%] predicted by the duration of the event [in hours] separated by event type (grooming N=31, resting N=18, intergroup encounter N=21). The effect of the interaction between Event and Duration is not significant (Wald test: *df=2, χ2=* 5.21, p=0.074).

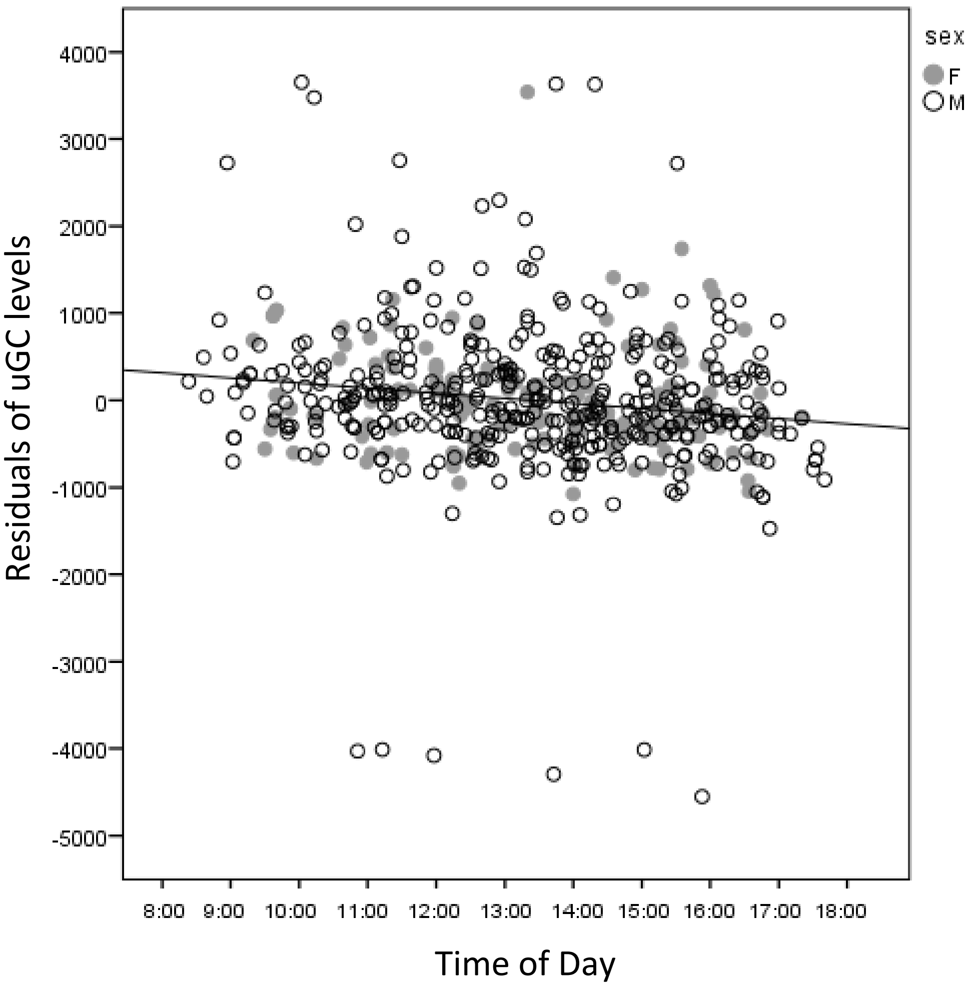

<u>Supplementary Figure 2:</u> Diurnal decrease of urinary glucocorticoid levels over time of day. Residuals of uGC levels from model regression line over time of day for Sonso chimpanzees (males: open circle; females: grey dot). Time of day significantly predicts uGC levels (Wald test: χ^2*=33.95*, *df*=1, *p*<0.001) with an average decline of 76.6 ng mg^{-1} creatinine $hour^{-1}$, as does Subject's Sex (Wald test: χ^2*=6.66* , *df*=1, *p*<0.01), with males showing higher levels than females. Number of urine samples N=574.

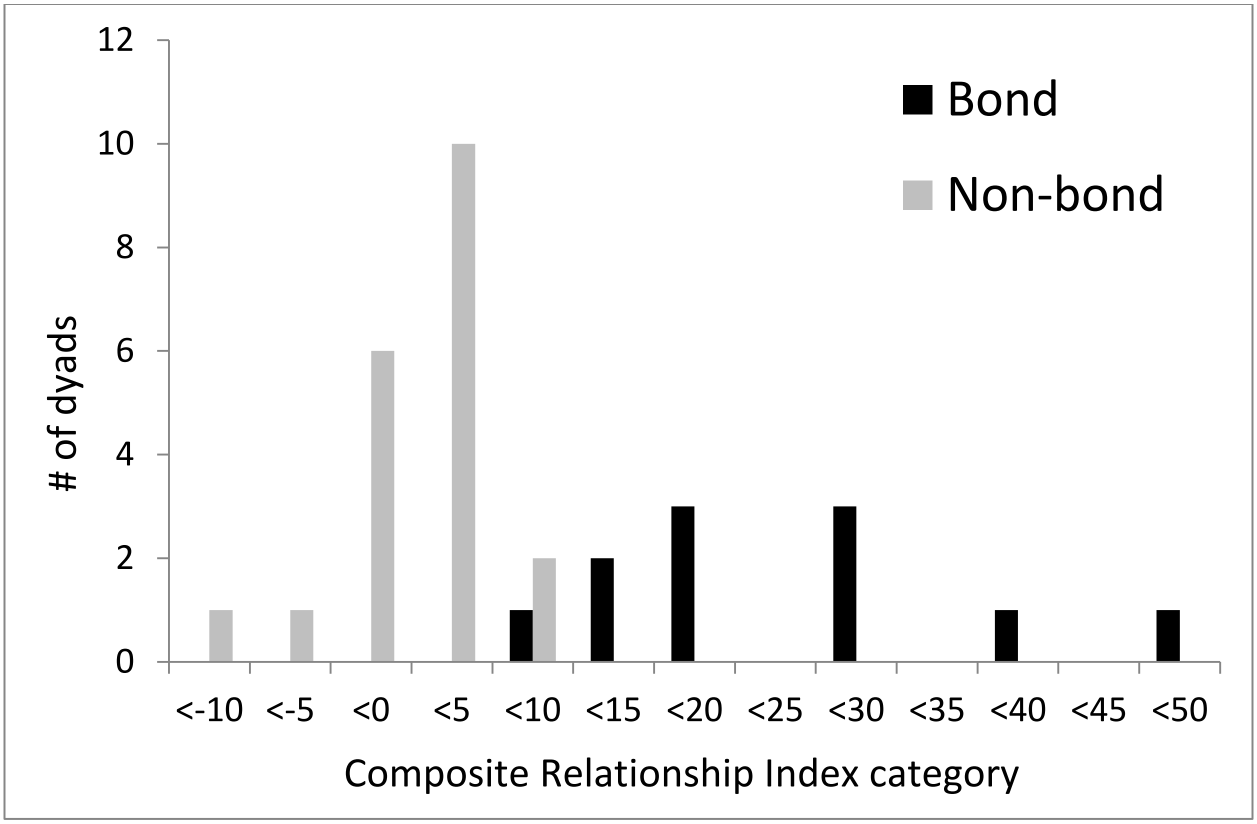

<u>**Supplementary Figure 3:**</u> **Bond and non-bond partners form two different distributions.** Relationship Quality shows a binomial distribution when plotted over the Composite Relationship Index (CRI).

Supplementary Table 1. Interaction of Event and Duration of Event does not significantly impact urinary glucocorticoid levels.

**Predictor variable**	***df***	***χ2***	***p***	**Parameter**	***β***	***SE***	***t***
				Intercept	64.4	13.9	
Event x	*2*	*5.2*	*0.074*	*Intergroup x duration*	*1.2*	*0.58*	*2.05*
Duration of event[#]				*Rest x duration*	*0.13*	*0.31*	*-0.43*
				Groom x duration	*0*		
Sex of subject[‡]	1	0.18	0.67	Male	-3.5	8.23	-0.43
				Female	0		
Number of Chimpanzees Present[‡]	1	5.2	0.66				

LMM: Likelihood ratio test (full vs. null model comparison): $\chi2=16.08$, $df=5$, $p=0.007$, [#]test predictor, [‡]control variable; Random factors: Identity of Subject, Event Number (more than one chimpanzee was sometimes sampled during the same event). Italics: $P < 0.1$. Intergroup: Intergroup encounter.

<u>Supplementary Table 2.</u> Result of the control model for simulated intergroup encounters (buttress drum experiments).

	**Intergroup encounters**
Response variable	Relative uGC level
Test predictor	Chimpanzees Present [#]
	Experiment [yes, no]
Control predictor	---
Random effect	Subject ID
	Event ID
Likelihood ration test	$\chi^2 = 0.869$, $df = 2$, $p = 0.648$

Neither the number of Chimpanzees Present nor whether the intergroup encounter was simulated or natural changed the relative uGC levels.

Supplementary Table 3. The impact of Event and Relationship Quality on urinary glucocorticoid (uGC) levels when comparing intergroup encounters against resting events (a) or against grooming events (b).

**Response variable:**	**relative uGC**						
**Predictor variable**	***df***	***χ2***	***p***	**Parameter**	***Estimate***	***SE***	***T***
**(a) Intergroup encounters vs. resting events**							
**(social buffering hypothesis)**				Intercept	103.40	20.66	
Event * Relationship Quality[#]	1	1.01	0.315	Resting * Bond	24.56	24.26	1.01
Kin[‡]	1	1.68	0.195	Kin	25.03	19.12	1.31
Subject's Sex * Partner's Sex[‡]	1	0.10	0.747	Male * Male	-9.26	28.70	-0,32
**(b) Intergroup encounters vs. grooming events**							
**(main effects hypothesis)**				Intercept	62.26	21.27	
**Event[#]**	**1**	**5.91**	**0.015**	**Intergroup**	**22.45**	**8.97**	**2.45**
**Relationship Quality[#]**	**1**	**6.84**	**0.009**	**Bond**	**-27.96**	**10.34**	**-2.7**
Kin[‡]	1	2.53	0.11	Kin	27.89	17.33	1.61
Subject's Sex[‡]	1	0.13	0.72	Male	4.68	12.97	0.36
**Partner's Sex[‡]**	**1**	**4.01**	**0.045**	**Male**	**28.87**	**14.14**	**2.04**

LMM: [#]test predictor, [‡]control variable; Random factors: Identity of Subject, Event. Bold: P < 0.05. Intergroup: Intergroup encounter. (a) Comparing intergroup encounters versus resting events testing social buffering hypothesis (Likelihood ratio test (full v null model comparison): *df=3, χ2=* 7.67, p=0.053). (b) Comparing intergroup encounters versus grooming events testing the main effects model after removing all non-significant interactions (Likelihood ratio test (full v null model comparison): *df=2, χ2=* 12.42, p=0.002; Effect size of fixed effects: marginal R^2=0.234).

Supplementary Note 1. Diurnal decrease of uGC levels in chimpanzees

The samples showed a mean uGC level ± SD [ng mg^{-1} creatinine] = 1382 ± 1152. We investigated whether or not uGC levels showed a diurnal distribution. We ran LMM procedures over all samples (N = 574) with log_{uGC} [ng mg^{-1} creatinine] as the response variable (logarithmic transformation of uGC levels due to lack of normal distribution of the residuals in the qq-plot) and subject identity as a random factor. We tested the effect of urination time [min after midnight] as a test variable and sex of subject [male, female] as a control variable. The full model better explained the variation of uGC levels than the null model, where predictor variables were removed. The results revealed that the uGC levels dropped significantly with 1.277 ng mg^{-1} creatinine $minute^{-1}$ (Supplementary Fig. 2) as time of day increased (time of day [min], Wald test: β=-0.001; $\chi^2=33.95$, df=1, p<0.001), and males had higher uGC levels than females (subject's sex [M,F], Wald test: β=0.513; $\chi^2=6.66$, df=1, p<0.01). In sum, GC concentrations from urine samples of both male and female chimpanzees displayed a clear diurnal pattern with lower concentrations occurring later in the day and an average decline of 76.6 ng mg^{-1} creatinine $hour^{-1}$, representing an average decline of 5.55% $hour^{-1}$. With an average delay of 2.25 hours between pre- and peak-samples (length of either period), we expect the peak-sample to be 12.5% lower than the pre-sample if there was no effect of the activity on the uGC level. Therefore we used an expected relative uGC level of 87.5% to compare with measured samples in the bootstrap resampling method.
